# Maternal Selenium-Enriched Yeast Supplementation in Sows Enhances Offspring Growth and Antioxidant Status through the Nrf2/Keap1 Pathway

**DOI:** 10.3390/antiox12122064

**Published:** 2023-12-01

**Authors:** Liang Xiong, Tongbin Lin, Xianhuai Yue, Shuchang Zhang, Xinghong Liu, Fang Chen, Shihai Zhang, Wutai Guan

**Affiliations:** 1 Guangdong Provincial Key Laboratory of Animal Nutrition Control, College of Animal Science, South China Agricultural University, Guangzhou 510642, China; 244628426@stu.scau.edu.cn (L.X.); 13616905519@stu.scau.edu.cn (T.L.); yxh@stu.scau.edu.cn (X.Y.); zsc0722@stu.scau.edu.cn (S.Z.); lxh@stu.scau.edu.cn (X.L.); chenfang1111@scau.edu.cn (F.C.); 2 National Engineering Research Center for Breeding Swine Industry, South China Agricultural University, Guangzhou 510642, China; 3 Guangdong Laboratory for Lingnan Modern Agriculture, South China Agricultural University, Guangzhou 510642, China

**Keywords:** redox status, sows, selenium-enriched yeast, piglets, small intestine, Nrf2/Keap1

## Abstract

This study evaluated the effects of maternal selenium-enriched yeast (SeY) supplementation during late gestation and lactation on sow performance, transfer of selenium (Se) and redox status, and gut microbiota community, as well as on the gut health of offspring. Seventy pregnant sows on day 85 of gestation were randomly allocated to the following two treatments: (1) sows who were fed a basal diet (basal diet contained 0.3 mg/kg Se as Na_2_SeO_3_, *n* = 35); (2) and sows who were fed a SeY-supplemented diet (basal diet with 0.2 mg/kg Se as SeY, *n* = 35). The offspring piglets were only cross-fostered within the group on day 3 of lactation (L3) according to the pig farm epidemic prevention policy. The plasma, milk, and feces samples from 10 sows, as well as plasma and intestinal samples per treatment, were collected on L1 and L21, respectively. Our results showed that maternal SeY supplementation increased the first week average weight and ADG of piglets (*p* < 0.05). Compared with the CON group, the SeY supplementation increased the Se content in the plasma and milk of sows and the plasma of piglets on L1 and L21 (*p* < 0.05). In addition, in sows, the levels of fat in the milk on L21, the level of IgA, T-AOC, and GSH-Px in the plasma on L21, and the level of T-AOC and GSH-Px in the colostrum were increased, while the MDA content was decreased in the plasma on L1 and in the colostrum and milk on L14 (*p* < 0.05). In the piglet plasma, the levels of IgA on L1 and L21, GSH-Px on L1, and GSH on L21 were increased, while the MDA content was decreased on L1 (*p* < 0.05). Maternal SeY supplementation up-regulated the small intestinal protein abundances of MUC1, E-cadherin, ZO-1, occludin, and claudin and activated the Nrf2/Keap1 signaling pathway in weaned offspring piglets. The 16S rRNA sequencing results showed that fecal microbiota had distinct separations during lactation, and the relative abundances of *unclassified_f_Lachnospiraceae*, *Prevotaceae_UCG-001*, and *Lachnospiraceae_NK4A136_group* were increased on L1. Collectively, the current findings suggest that maternal SeY supplementation during late gestation and lactation could improve the piglet’s growth performance, Se status, antioxidant capacity and immunoglobulins transfer at the first week of lactation, as well as alter the fecal microbiota composition by increasing antioxidative-related and SCFA-producing microbiota in sows. These changes contributed to enhancing the small intestinal barrier function and activating the Nrf2/Keap1 pathway in offspring.

## 1. Introduction

Genetic selection for hyperprolific sows suffers from highly stressful conditions including increased milk production, limited nutrient intake, and increased oxidative stress [1]. Studies have shown that reactive oxygen species (ROS) are increased in late pregnancy and lactation, and maternal oxidative stress may transmit to offspring [2,3]. Early-weaning piglets are met with continuing external stressors, including changing dietary habits, gut microbiota, and environmental factors, leading to intestinal oxidative injury and dysfunction [4]. Intestinal oxidative injury commonly results in a low feeding efficiency and growth performance in piglets [5]. Thus, the oxidative stress during the neonatal and weaning phases may continuously lead to a long-term negative effect on the growth performance, which can persist until adulthood [6]. Previous studies have indicated that antioxidant protection and immune agents, achieved via nutrients, might be transferred from the mother to the newborn during pregnancy or via the maternal colostrum and milk supply [7,8]. Our previous studies have shown that maternal feed additives could affect the offspring’s intestinal function through regulation of signal pathways [9]. Therefore, the redox status homeostasis of sows during late gestation and lactation would be of great significance for their reproductive performance and for offspring gut development.

Selenium (Se) is an indispensable trace element to sows and exists in nature in both inorganic and organic forms [10]. Se is a metalloid that is incorporated into polypeptide chains as part of the selenocysteine to fulfil a biofunction. The proteins that contain selenocysteine in their poly-peptide chain are identified as selenoproteins [11]. The major selenoprotein glutathione peroxidase (GSH-Px) enzyme is well known due to its role in the detoxification of hydrogen peroxide and lipid hydroperoxides inside the cellular membrane against ROS damage [12]. The Se content in a sow’s blood continues to decrease as the pregnancy and lactation progresses [13]. A Se deficiency during gestation leads to miscarriage, pre-eclampsia, preterm birth, and intrauterine growth retardation [11]. Compared with inorganic forms of Se, selenium-enriched yeast (SeY) mainly consists of selenoamino acids (i.e., selenomethionine) and their analogues [14]. As the organic form of Se, SeY is primarily used on account of the more efficient deposition in maternal tissues and transfer to offspring [15,16,17]. Hence, it is crucial to supplement SeY during late gestation and lactation in sow diets for the redox status of sow and progeny.

Previous studies showed that when Se was supplied in an organic instead of inorganic form in a sow diet during gestation and lactation, it could improve the reproductive performance and the Se and redox status in the serum, milk, and offspring [13,18,19]. However, other studies have reported that different dietary Se sources or levels when fed during gestation and lactation had no effect on the reproductive performance, including lactation feed intakes, parturition performance, and litter performances of sows [15,16,20]. Our previous study also reported that increasing the SeY supply during late gestation did not affect the reproductive performance of sows [21]. The studies that refer to the effect of SeY supplementation during late gestation and lactation on the reproductive performance of sows are inconsistent [22]. Moreover, the effect of maternal SeY supplementation during late gestation and lactation on the sow’s reproductive performance, transfer of selenium and redox status, and gut microbiota community, as well as on the intestinal health of the offspring, has not been extensively studied. Therefore, we investigated the effect of maternal SeY supplementation during late pregnancy and lactation on the sow performance, transfer of redox status, and the intestinal expression of the nuclear factor erythroid2-related factor (Nrf2) and Kelch-like ECH-associated protein 1 (Keap1) pathway in the offspring. We hypothesized that maternal SeY supplementation during late gestation and lactation could improve the lactation performance, antioxidant capacities in sows and their offspring, and gut health through the Nrf2/Keap1 signal pathway. The current study was conducted to verify this hypothesis.

## 2. Materials and Methods

### 2.1. Animals, Treatments, and Diets

A total of 70 multiparous (3 to 4 parities) Landrace × Large White sows were randomly selected from a commercial herd located in Guangdong Foodstuffs IMP. & EXP. Group Co., Ltd. in Heyuan City, Guangdong Province, China. Subsequently, the sows were randomly allocated to 2 treatments (35 sows per group) by parity, body condition, and historical reproductive performance as follows: (1) CON group, basal diet contained 0.3 mg/kg Se as Na_2_SeO_3_, *n* = 35 (this level is industry level, above recommendation level); (2) SeY group, basal diet with 0.2 mg/kg Se as Se-enriched yeast, *n* = 35 (Se-enriched yeast with 2000 mg/kg Se; Sel-PlexTM 2000, Alltech Inc., Lexington, KY, USA). The Se level in SeY group did not exceed the upper limit level (0.5 mg/kg) of Se in Food and Drug Administration (FDA) standards in the USA and standards in China. The nutrition levels of the basal diets met and exceeded the nutritional requirements of pregnant and lactating sows according to the National Research Council (NRC) (2012). No antibiotics were used during the entire trial. The feed composition and nutrient level of the basal diet are listed in Table S1.

All sows were housed in individual pregnancy crates (2.2 × 0.8 m) from day 85 until 106 of gestation; then, they were transferred into the parturition rooms on day 107 of gestation until day 23 of lactation, and the total trial period was 52 days. Sows were fed twice per day at 700 and 1600 h. Sows were restricted to feed and received a total of 3.0–3.5 kg/d diets per sow. The farrowing facility where the sows were placed in individual delivery crates (2.5 × 2.5 m) had environment-controlled systems. The room temperature of farrowing rooms was always kept at 21–23 °C. On the farrowing day, sows were fed 1.0 kg of diet and the amount of feed gradually increased by 1.0 kg/d until feeding ad libitum. At farrowing, the total number of piglets born, born alive, stillborn, as well as neonatal piglets’ weight (before colostrum intake) was recorded. According to the pig farm epidemic prevention policy, piglets were only cross-fostered within the group on day 3 of lactation; then, the litter size was standardized to 10–12 piglets per litter. Animals had access to water ad libitum throughout the trial. Piglets were weaned on day 23 of lactation and had no access to creep feed. During lactation, feed intake of each sow was recorded to calculate ADFI. In addition, the individual piglet’s body weight (BW) and litter size were recorded after being cross-fostered on day 3 of lactation and at weaning on day 23 of lactation. Using these data, the average daily gain (ADG) and survival rate during lactation of piglets were calculated. The weaning-to-estrus interval of sows was tracked and recorded after the weaning of offspring. Backfat thickness was determined at the P2 position (6 cm away from the spine and at the head of the last rib) using an ultrasonic device (Renco Lean-Meater^®^, Renco Corporation, Minneapolis, MN, USA) on day 110 of gestation and day 23 of lactation to assess the backfat loss of sows during lactation.

### 2.2. Sample Collection

#### 2.2.1. Collection of Feed and Feces of Sows

The 10 sows per group that were selected for sample collection on day 1 and 21 of lactation were always the same. Approximately 1 kg of pregnancy and lactation diets were collected and stored at −20 °C weekly. On day 1 and 21 of lactation, 10 sows per group were selected for collection of fresh fecal samples through the rectal palpation method, and samples were frozen at −80 °C immediately.

#### 2.2.2. Collection of Plasma Samples of Sows and Piglets

The 10 sows and 6 offspring piglets per group that were selected for sample collection on day 1 and 21 of lactation were always the same. Blood samples (5 mL) were obtained from 10 sows per group via the ear vein and 6 offspring piglets per group via the precaval vein using heparinized vacutainer tubes on day 1 and 21 of lactation. The blood samples were centrifugated at 3000× *g* for 15 min to acquire plasma samples, which were deposited at −20 °C for subsequent analysis.

#### 2.2.3. Collection of Colostrum and Milk of Sows

After delivery, approximately 20 mL of colostrum samples were collected immediately from every functional teat (10 sows per group) on day 1 of lactation. After injection of 3 mL oxytocin, approximately 20 mL milk samples were collected on day 14 and 21 of lactation. The colostrum and milk samples were immediately frozen at −80 °C for further analysis.

#### 2.2.4. Collection of Small Intestinal Mucosa of Piglets

On day 23 of lactation, one piglet with close to the average BW of each litter (*n* = 6 per treatment) was selected and euthanized humanely after weaning. The entire intestine was removed from the abdominal cavity. After removal of digesta, the segments (2 cm) were collected from the middle duodenum, jejunum, and ileum, and then fixed in 20% paraformaldehyde. And then, the mucosa samples of small intestine were scraped using a piece of glass, then quickly snap-frozen in liquid nitrogen and stored at −80 °C for subsequent protein extraction.

### 2.3. Laboratory Analysis

#### 2.3.1. Analysis of Selenium Content in Feed, Plasma, and Milk

The measurement of Se content in feed, plasma, and milk was conducted using fluorometric method outlined by the “National Food Safety Standard Determination of Selenium in Food” (GB5009.93-2017) [23], which was referred to in our previous studies [21]. The Se standard was purchased from SCP Science (Cat#: 140-051-340, Montreal Island, QC, Canada). First, 1 g diet or 1 mL plasma and milk samples were digested using complex acids (the ratio of nitric acid and perchloric acid was 4 to 1) for overnight digestion at room temperature. Se complexes in diet and blood samples were oxidized to be Se^4+^, which immediately reacted with 2,3-diaminonaphthalene to form 4,5-benzo piaselenol in acidic medium, and then was extracted using cyclohexane. Subsequently, the fluorescence of the organic phase was analyzed via fluorescence spectrometry (RF-5301, shimadzu corporation, Kyoto, Japan). The Se content has a linear correlation with fluorescence degree when Se content in samples is less than 0.5 μg.

#### 2.3.2. Analysis of Colostrum and Milk Composition

Colostrum and milk samples (5 mL per sow) were assessed to determine the contents of solids-not-fat, fat, protein, and lactose using a fully automatic milk composition analyzer (MilkoScan^®^ FTp Analyzer, Foss, Hillerød, Denmark).

#### 2.3.3. Analysis of Immune and Redox Status in Plasma and Milk

Concentrations of immunoglobulin A (IgA), IgG, IgM in plasma, colostrum, and milk were determined using ELISA kits according to the manufacturer’s protocols (Huamei Biotechnology, Wuhan, China). In addition, the levels of the total antioxidant capacity (T-AOC), total superoxide dismutase (T-SOD), glutathione (GSH), GSH-Px, and malondialdehyde (MDA) in plasma, colostrum, and milk were measured using commercial ELISA kits according to the manufacturer’s instructions (Nanjing Institute of Jiancheng Biological Engineering, Nanjing, China).

#### 2.3.4. Analysis of Relative Protein Abundances

Small intestine tissue proteins were extracted via RIPA Lysis buffer (Beyotime, Jiangsu, China), which added a 1% protease inhibitors PMSF and 1% phosphatase inhibitors cocktail. The concentration of total protein was measured using the BCA assay kit (Beyotime, Jiangsu, China). Resolution of protein was determined via sodium dodecyl sulfate-polyacrylamide gel electrophoresis (SDS-PAGE) gel, followed by a transfer to polyvinylidene difluoride (PVDF) membranes and nonspecific bind blocking with 5% BSA. The membranes were incubated with the probed primary antibodies against β-actin (#4970, CST, Hong Kong, China), Claudin1 (#ab211737, Abcam, Cambridge, UK), Occludin (#ab216327, Abcam), Zonula Occludens (ZO)-1 (#21773-1-AP, Proteintech, Rosemont, IL, USA), Mucin (MUC)1 (#14161, CST), Nrf2 (#ab62352, Abcam), p-Nrf2 (bs-2013R, Bioss, Woburn, MA, USA), and Keap1 (#8047, CST) at 4 °C overnight. After that, membranes were washed with TBST (phosphate-buffered saline with Twen-20) buffer and incubated with a suitable secondary antibody for 1.5 h at room temperature. Finally, target bands were visualized using the Chemiluminescence Image Analysis System (Tanon, Shanghai, China). The relative abundances of target proteins were normalized against β-actin and quantitated using the ImageJ software 1.33 (National Institutes of Health, Bethesda, MD, USA).

#### 2.3.5. Immunofluorescence Analysis of Small Intestine

The distribution and abundance of MUC1 protein in the small intestine of the weaned offspring piglets were determined using immunofluorescence. The small intestine tissues were immobilized and fixed with paraffin wax, and 5 µm sections were prepared. Subsequently, the sections were blocked with 3% bovine serum albumin and incubated overnight at 4 °C with rabbit anti-MUC1 primary antibody (#ab451671, 1: 200; Abcam, Cambridge, UK). Then, the sections were washed five times with phosphate-buffered salt solution (PBS) and incubated with goat antirabbit fluorescein isothiocyanate (FITC)-conjugated secondary antibody (#bs-0295G, Bioss, Beijing, China) for 40 min at room temperature in the dark. Finally, the sections were washed five times with PBS again, and the cell nuclei were stained with 4′-6-diamidino-2-phenylindole (DAPI, #0100-01, SouthernBiotech, Birmingham, AL, USA) for 30 min at room temperature in the dark. Fluorescence imaging of each section was performed using a fluorescent scanning microscope (Nikon Eclipse TI-SR, Tokyo, Japan), and images were captured with the NIKON DS-U3 software 3.0.

#### 2.3.6. Analysis of Fecal Microbiota of Sows

Total bacterial genomic DNA was extracted from 100 mg fecal samples of sows on day 1 and 21 of lactation using the E.Z.N.A.^®^ soil DNA Kit (Omega Bio-tek, Norcross, GA, USA) according to manufacturer’s instructions. The quality and content of DNA were detected using 1.0% agarose gel electrophoresis, quantitated via NanoDrop2000 spectrophotometer (Thermo Scientific, Waltham, MA, USA), and then stored at −80 °C for subsequent analysis. The V3–V4 region of the microbial 16S rRNA gene was amplified with primer 338F (5′-ACTCCTACGGGAGGCAGCAG-3′) and 806R(5′-GGACTACHVGGGTWTCTAAT-3′) pairs using a T100 Thermal Cycler PCR thermocycler (BIO-RAD, Hercules, CA, USA). The final volume of 20 µL PCR reaction mixture consisted of 4 μL 5× Fast Pfu buffer, 2 μL 2.5 mM dNTPs, 0.8 μL of each primer (5 μM), 0.4 μL Fast Pfu polymerase, 10 ng of template DNA, and ddH_2_O. The amplicon libraries were sequenced on the Illumina MiSeq platform (Illumina, San Diego, CA, USA) using the MiSeq Reagent Kit v3 600 cycles. The sequencing was performed according to the standard protocols of Shanghai Majorbio Biotechnology Co., Ltd. (Shanghai, China).

Pan analysis at the operational taxonomic unit (OTU) level of fecal samples between two groups was performed, evaluating whether the sequencing depth was sufficient to cover all OTUs according to whether the Pan species curve is flat or not. Alpha diversity indices (Ace and Chao1 richness; Shannon and Simpson) of all OTUs were measured using the OTU table and the QIIME2 (2019.4) software. The beta diversity analysis was performed to evaluate the structural variation in fecal microbiota communities. Principal component analysis (PCA) and partial least squares discriminant analysis (PLS-DA) were jointly carried out to determine the significant differences in microbial communities between the two groups. The differences in the relative abundances of fecal microbiota at the phylum and genus levels were simultaneously compared using STAMP 2.1.3 software. The biomarkers of the microbial taxa were identified using the linear discriminant analysis (LDA) effect size (LEfSe) (https://huttenhower.sph.harvard.edu/galaxy/ (accessed on 5 September 2023)), and the graph was plotted with a default parameter LDA score > 2.0.

### 2.4. Statistical Analyses

Statistical differences of two groups were assessed via Student’s *t*-test using the SPSS 22.0 software (Chicago, IL, USA). Each sow (or litter), for reproductive performance analysis, and individual piglets (per litter) for other parameter analyses were used as the experimental unit, respectively. All data are expressed as means ± standard error of the mean (SEM). Statistical difference value between the two treatments was considered significant with *p* < 0.05, and 0.05 < *p* < 0.10 was set as a trend toward difference. The differences in alpha diversity indices and the relative microbial abundances were analyzed using the Mann–Whitney U-test. A corrected *p* value < 0.05 was considered statistically significant.

## 3. Results

### 3.1. Se Transfer in Diets, Sows, and Offspring

**Figure 1:**
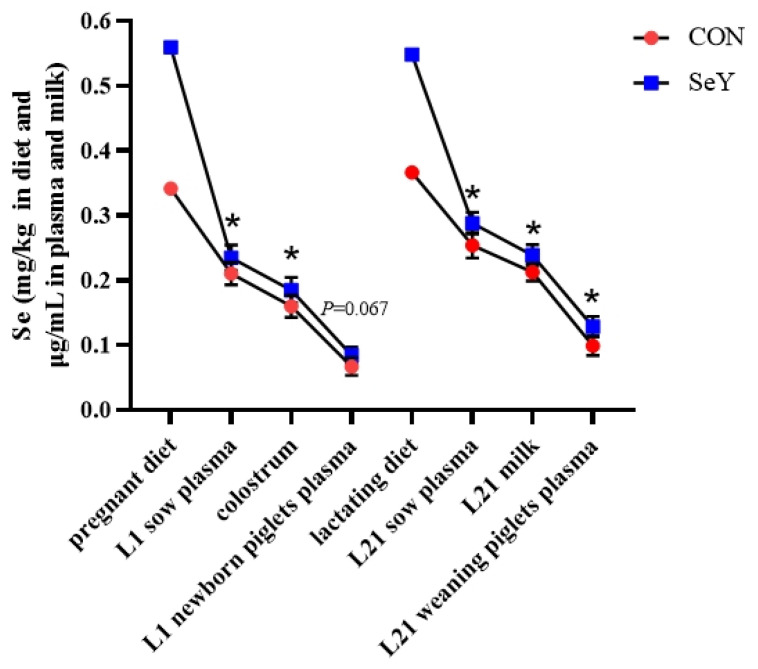
Effect of maternal SeY supplementation during late gestation and lactation on Se transfer from sow diets to offspring piglets. Data are represented as means ± SEM, *n* = 6 per group, * *p* < 0.05. CON = control group, sows fed basal diet containing 0.3 mg/kg Se as Na_2_SeO_3_; SeY = SeY group, sows fed basal diet with 0.2 mg/kg Se as Se-enriched yeast (Se-yeast with 2000 mg/kg Se).

The Se content in pregnancy and lactation experimental diets as well as the Se transfer from sows to offspring piglets are shown in Figure 1. The pregnancy diets were determined to contain 0.34 and 0.56 mg Se/kg diet in the CON and SeY groups, respectively. The lactation diets were determined to contain 0.37 and 0.55 mg Se/kg diet in the CON and SeY groups, respectively. A higher Se content in sows’ and offspring’s plasma and sow milk on day 1 and 21 of lactation was detected in the SeY group compared with those in the CON group (*p* < 0.05).

### 3.2. The Reproductive Performance of Sows

The sows’ reproductive performance is presented in Table 1. The results showed that SeY supplementation during late gestation and lactation showed no effect on the number of piglets born in total, born alive, stillbirths, mummies, and born healthy of sows (*p* > 0.05). In addition, there was no evidence of differences between the two groups in terms of litter weight at birth, litter birth weight, individual piglet weight, weight when born healthy, stillborn weight, and number of pigs weaned, for which the differences were small and insignificant (*p* > 0.05). No significant treatment differences were observed for placental weight, placental weight per piglet, or placental efficiency (*p* > 0.05).

### 3.3. Lactation and Litter Performance of Sows

The effect of maternal SeY addition during late gestation and lactation on the growth performance of nursing piglets is presented in Table 2. The results showed that maternal SeY supplementation during late gestation and lactation had no effect on the litter size and litter weight during the lactation (*p* > 0.05). Maternal SeY supplementation during late gestation and lactation significantly increased the final average weight and mean ADG of offspring piglets in the first week (*p* < 0.05).

### 3.4. Feed Intake, Backfat Thickness, and Weaning-to-Estrus Interval of Sows

The feed intake, backfat thickness, and weaning-to-estrus interval of sows are shown in Table 3. No significant treatment differences for ADFI or backfat thickness were observed between the two treatments of sows during the entire lactation (*p* > 0.05). Compared with the CON group, the weaning-to-estrus interval was significantly declined in the SeY group (*p* < 0.05).

### 3.5. Colostrum and Milk Composition

The compositions of the colostrum and milk are presented in Table 4. There were insignificant differences between the two groups in the composition of colostrum with regard to fat, lactose, protein, and solids-not-fat in the colostrum and milk on day 14 of lactation (*p* > 0.05). Compared with the CON group, the SeY supplementation increased the fat content in the milk on day 21 of lactation (*p* < 0.05).

### 3.6. Immune Indicators in the Plasma and Milk of Sows

The immunoglobulin concentrations in the plasma and milk of sows are shown in Table 5. In the plasma, the IgA contents on day 1 and 21 of lactation were significantly higher in the SeY group than those in the CON group (*p* < 0.05). In the milk, the IgG contents on day 14 and 21 of lactation also showed an up-regulated trend in the SeY group (*p* = 0.076, *p* = 0.059).

### 3.7. Immune Indicators in the Plasma of Offspring Piglets

The immunoglobulin concentrations in the plasma of the offspring piglets are shown in Table 6. In the plasma, the IgA contents at 1 and 21 days of age were also significantly higher in the SeY group than those in the CON group (*p* < 0.05), and an up-regulated trend of the IgM content was observed in the SeY group of piglets at 1 day of age (*p* = 0.061).

### 3.8. Antioxidative and Oxidative Indicators in the Plasma and Milk of Sows

The oxidative and antioxidative indexes of the plasma and milk of sows are shown in Table 7. In the plasma, the levels of T-AOC and GSH-Px were increased on day 21 of lactation (*p* < 0.05), whereas the MDA concentration was decreased on day 1 of lactation in the SeY group compared with the CON group (*p* < 0.05). In the colostrum and milk, the levels of T-AOC and GSH-Px in the colostrum and T-SOD in milk on day 21 of lactation were increased (*p* < 0.05), while the MDA concentrations in the colostrum and, on day 14 of lactation, the milk were decreased in the SeY group compared with the CON group (*p* < 0.05).

### 3.9. Antioxidative and Oxidative Indicators in the Plasma of Offspring Piglets

The oxidative and antioxidative indexes of the plasma of offspring piglets are shown in Table 8. Compared with the CON group, the content of T-SOD at 1 day of age and the levels of T-AOC and GSH at 21 days of age were significantly raised (*p* < 0.05), while the level of MDA was significantly reduced at 1 and 21 days of age in the SeY group (*p* < 0.05).

### 3.10. The Small Intestinal Barrier Function of Offspring Piglets

**Figure 2:**
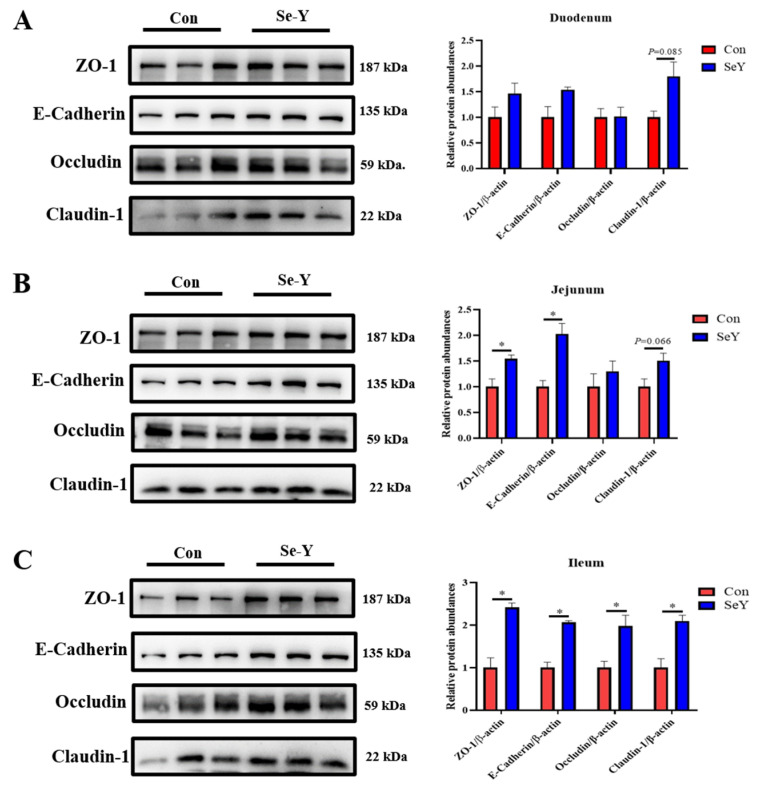
Effects of maternal SeY supplementation during late gestation and lactation on the relative abundances of tight junction (TJ) protein in the duodenum (**A**), jejunum (**B**), and ileum (**C**) of weaned offspring piglets. Data are represented as means ± SEM, *n* = 6 per group, * *p* < 0.05. CON = control group, sows fed basal diet containing 0.3 mg/kg Se as Na_2_SeO_3_; SeY = SeY group, sows fed basal diet with 0.2 mg/kg Se as Se-enriched yeast (Se-yeast with 2000 mg/kg Se).

**Figure 3:**
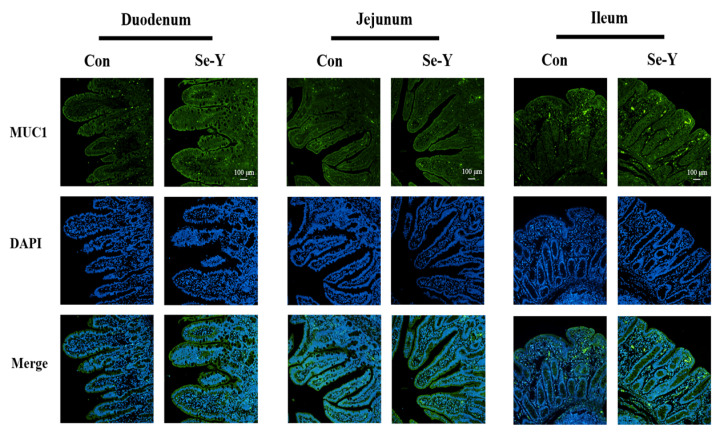
Representative micrographs for mucin (MUC)-1 staining in the small intestine tissues of offspring piglets under a confocal microscope. CON = control group, sows fed basal diet containing 0.3 mg/kg Se as Na_2_SeO_3_; SeY = SeY group, sows fed basal diet with 0.2 mg/kg Se as Se-enriched yeast (Se-yeast with 2000 mg/kg Se).

The effect of maternal SeY supplementation during late gestation and lactation on the small intestinal tight junction (TJ) proteins and mucus barrier of weaned offspring piglets are shown in Figure 2 and Figure 3. Compared with the CON group, maternal SeY supplementation tended to up-regulate the relative protein abundances of claudin-1 in the duodenum (*p* = 0.085) and jejunum (*p* = 0.066); ZO-1 (*p* < 0.05) and E-cadherin (*p* < 0.05) in the jejunum; and ZO-1 (*p* < 0.05), E-cadherin (*p* < 0.05), occludin (*p* < 0.05), and claudin-1 (*p* < 0.05) in the ileum of the weaned piglets (Figure 2). Moreover, the abundance and distribution of MUC1, the major mucus barrier protein, were investigated via immunofluorescence analysis. The localization of MUC1 in the small intestine of offspring piglets was not affected by SeY supplementation (Figure 3); however, maternal SeY supplementation significantly increased the MUC1 expression in the apical intercellular region of the small intestinal epithelium in piglets.

### 3.11. The Expression of Nrf2/Keap1 Pathway-Related Proteins in the Small Intestine of Suckling Offspring Piglets

**Figure 4:**
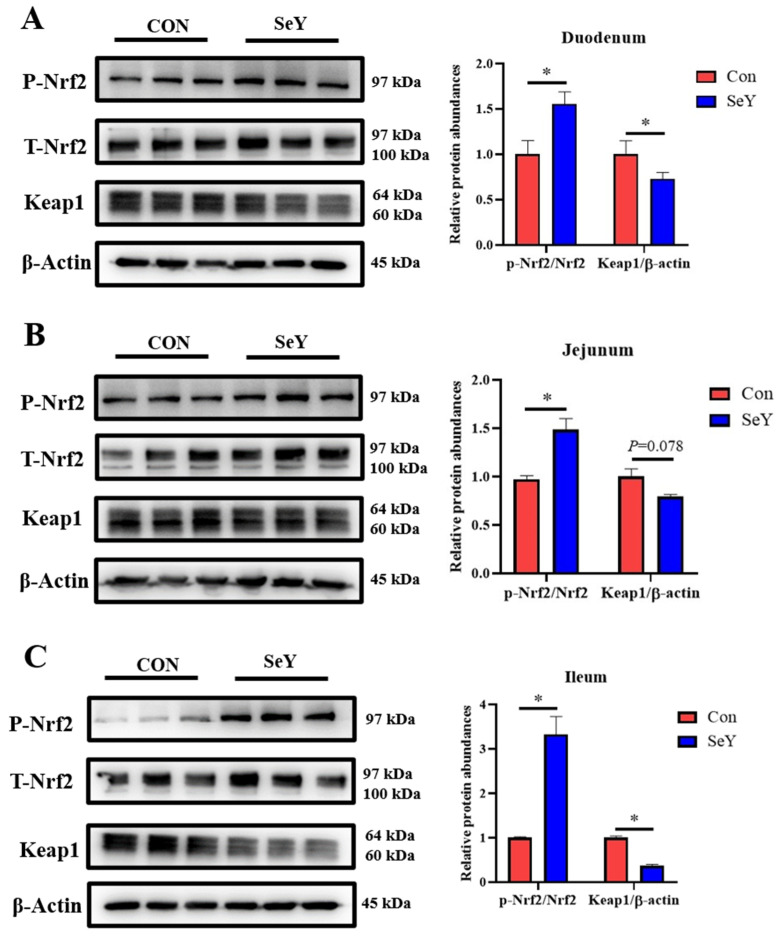
Effects of maternal SeY supplementation on the relative protein abundances involved in the Nrf2/Keap1 signaling pathway in the duodenum (**A**), jejunum (**B**), and ileum (**C**) of suckling offspring piglets. Data are represented as means ± SEM, *n* = 6 per group, * *p* < 0.05. CON = control group, sows fed basal diet containing 0.3 mg/kg Se as Na_2_SeO_3_; SeY = SeY group, sows fed basal diet with 0.2 mg/kg Se as Se-enriched yeast (Se-yeast with 2000 mg/kg Se).

The effect of maternal SeY supplementation during late gestation and lactation on the small intestinal Nrf2/Keap1 signaling pathway of weaned offspring piglets is shown in Figure 4. Compared with the CON group, maternal SeY supplementation significantly up-regulated the ratio of phosphorylated Nrf2 and total Nrf2 in the small intestine (*p* < 0.05), down-regulated the relative protein abundance of Keap1 in the duodenum (*p* < 0.05), and tended to increase in the jejunum (*p* = 0.078) and ileum (*p* < 0.05) of the weaned piglets.

### 3.12. Fecal-Associated Microbiota

**Figure 5:**
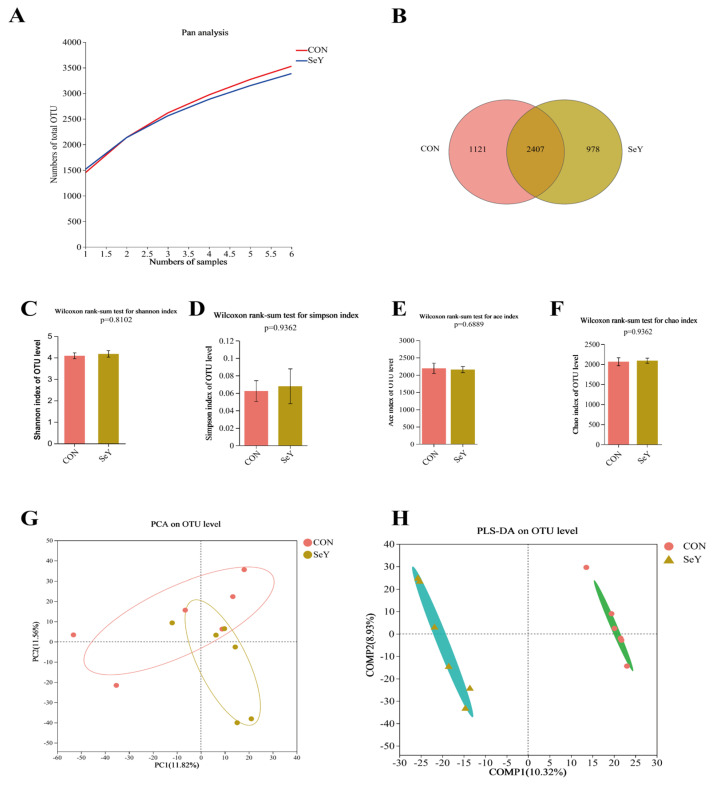
Effects of dietary SeY supplementation on the composition and structure of fecal microbiota in sows on day 1 of lactation. The Pan species curve of the sequencing depth of sows (**A**). The Venn diagram of OTU network analysis (**B**). The fecal microbial alpha diversity of sows, including Shannon (**C**), Simpson (**D**), Ace (**E**), and Chao1 (**F**) indexes. The principal component analysis (PCA) plot (**G**) and partial least squares discrimination analysis (PLS-DA) based on an unweighted UniFrac distance score plot (**H**) of microbial communities. *n* = 6 per group. CON = control group, sows fed basal diet containing 0.3 mg/kg Se as Na_2_SeO_3_; SeY = SeY group, sows fed basal diet with 0.2 mg/kg Se as Se-enriched yeast (Se-yeast with 2000 mg/kg Se).

**Figure 6:**
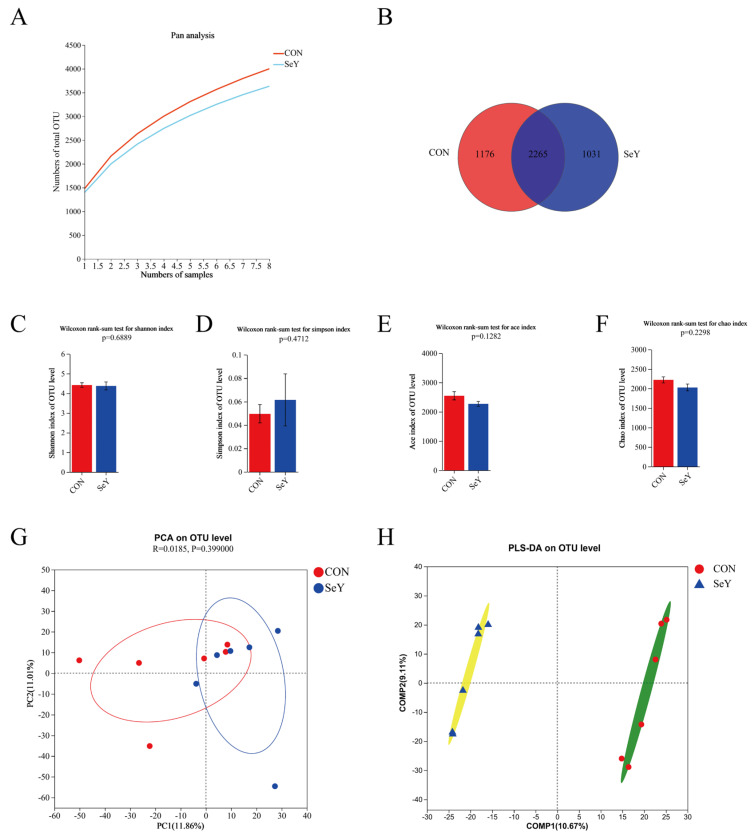
Effects of dietary SeY supplementation on the composition and structure of fecal microbiota in sows on day 21 of lactation. The Pan species curve of sequencing depth of sows (**A**). The Venn diagram of OTU network analysis (**B**). The fecal microbial alpha diversity of sows, including Shannon (**C**), Simpson (**D**), Ace (**E**), and Chao1 (**F**) indexes. The principal component analysis (PCA) plot (**G**) and partial least squares discrimination analysis (PLS-DA) based on an unweighted UniFrac distance score plot (**H**) of microbial communities. *n* = 6 per group. CON = control group, sows fed basal diet containing 0.3 mg/kg Se as Na_2_SeO_3_; SeY = SeY group, sows fed basal diet with 0.2 mg/kg Se as Se-enriched yeast (Se-yeast with 2000 mg/kg Se).

To understand the effects of dietary SeY supplementation during late gestation and lactation on the composition and structure of the fecal microbiota of sows, 16S rRNA sequencing of fecal samples on day 1 and 21 of lactation was performed. The Pan species curve is flat, indicating that the sample size for this sequencing is sufficient, and almost all bacterial species were captured from feces of sows on day 1 (Figure 5A) and 21 (Figure 6A) of lactation. The Venn diagram showed the OTU network analysis of the fecal microbiota of sows between the two groups on day 1 (Figure 5B) and 21 (Figure 6B) of lactation. A total of 3528 and 3385 microbial OTUs were detected, of which 1121 (31.77%) and 978 (28.89%) individual core microbial OTUs were shared on day 1 of lactation. In addition, a total of 3441 and 3296 microbial OTUs were detected, of which 1121 (31.77%) and 978 (28.89%) individual core microbial OTUs were shared on day 21 of lactation. The microbial alpha diversity indexes of the sow feces, including Ace, Chao1, Simpson, and Shannon indexes, are shown in Figure 5 and Figure 6. The alpha diversity showed no significant differences between the two groups on day 1 (Figure 5C–F) and 21 (Figure 6C–F) of lactation (*p* > 0.05). PCA and PLS-DA analyses were performed to distinguish the differences in the microbial community between the two groups. The microbial beta diversity results showed that there were distinct separations in the microbial OUT levels between the two groups on day 1 (Figure 5G,H) and 21 (Figure 6G,H) of lactation.

**Figure 7:**
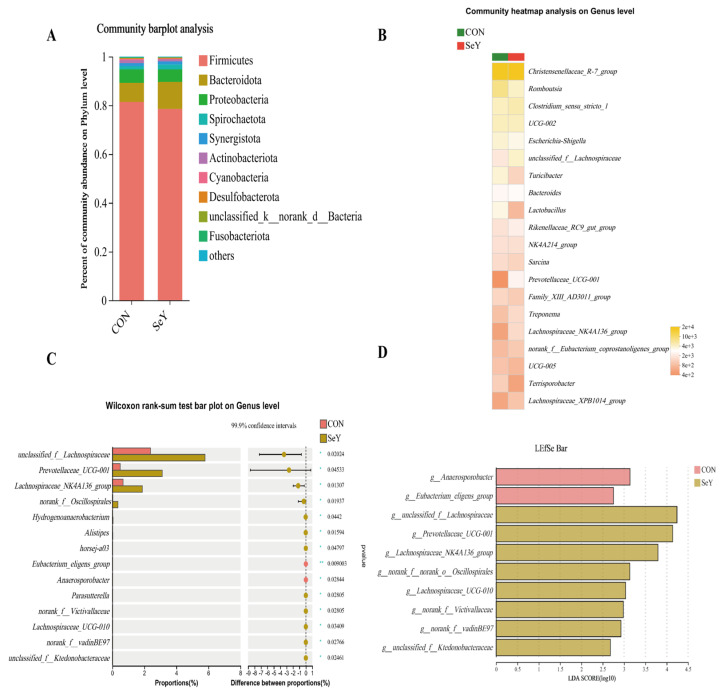
The fecal microbial communities and differential microbial biomarkers based on the relative abundance at phylum and genus levels of sows on day 1 of lactation. The average relative abundances of phylum level greater than 0.1% proportion (**A**) and genus level for the top 20 relative abundance (**B**) were listed. *n* = 6 per group, * *p* < 0.05, ** *p* < 0.01. The relative abundance differences analyzed via STAMP (**C**). Differential microbial biomarkers based on the relative abundance, filtrated via LEfSe analysis (**D**). CON = control group, sows fed basal diet containing 0.3 mg/kg Se as Na_2_SeO_3_; SeY = SeY group, sows fed basal diet with 0.2 mg/kg Se as Se-enriched yeast (Se-yeast with 2000 mg/kg Se).

**Figure 8:**
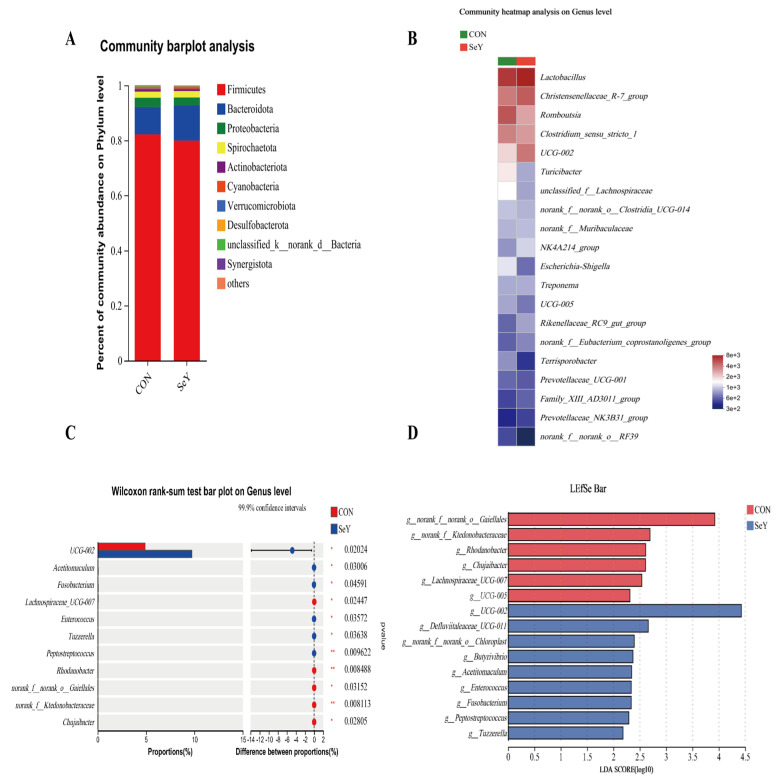
The fecal microbial communities and differential microbial biomarkers based on the relative abundance at phylum and genus levels of sows on day 21 of lactation. The average relative abundances of phylum level greater than 0.1% proportion (**A**) and genus level for the top 20 relative abundance (**B**) were listed. *n* = 6 per group, * *p* < 0.05, ** *p* < 0.01. The relative abundance differences analyzed via STAMP (**C**). Differential microbial biomarkers based on the relative abundance, filtrated via LEfSe analysis (**D**). CON = control group, sows fed basal diet containing 0.3 mg/kg Se as Na_2_SeO_3_; SeY = SeY group, sows fed basal diet with 0.2 mg/kg Se as Se-enriched yeast (Se-yeast with 2000 mg/kg Se).

The fecal microbial communities and differential microbial biomarkers based on the relative abundance of sows on day 1 and 21 of lactation at the phylum and genus levels are displayed in Figure 7 and Figure 8, respectively. At the phylum level, the top four dominant phyla were commonly identified in the two groups of fecal microbiotas of sows on day 1 and 21 of lactation: Firmicutes, Bacteroidota, Proteobacteria, and Spirochactota. In addition, the abundance distribution in the top 20 most abundant fecal microbiota of sows on day 1 (Figure 7B) and 21 (Figure 8B) of lactation at the genus level was plotted as a heatmap. Heatmaps were made using the vegan package in R (v3.3.1). Differences in the relative abundances of the community components of fecal microbiota of sows between the CON and SeY groups were further analyzed using the Mann–Whitney U-test via STAMP software 2.1.3. In addition, the relative abundance differences analyzed via STAMP and LEfSe analyses were performed together to detect the differential marker bacterium. The linear discriminant analysis (LDA) effect size (LEfSe) (http://huttenhower.sph.harvard.edu/LEfSe (accessed on 5 September 2023)) was calculated to identify differentially abundant bacterium (phylum to genus level) among the different samples (LDA score > 2.0, *p* < 0.05). At the phylum level, the STAMP results and LEfSe analysis together showed no significant difference between the two groups on day 1 and 21 of lactation. At the genus level, compared with the CON group, the SeY group had a significant increase in the relative abundances of *unclassified_f__Lachnospiraceae*, *Prevotellaceae_UCG-001*, *Lachnospiraceae_NK4A136_group*, and *norank_f_ Oscillospirales* on day 1 (Figure 7C,D) of lactation, and there was a significant decrease in the UCG-002 of sows on day 21 (Figure 8C,D) of lactation.

## 4. Discussion

Maternal nutrition is a predominant factor in regulating the redox status, immunity, and gut health of offspring piglets [24]. Thus, this study evaluated the effect of maternal SeY supplementation during late gestation and lactation on sow performance, transfer of Se and antioxidant capacity, and gut microbiota community, as well as on the intestinal health of offspring. Consistent with our experimental hypothesis, the findings in the present study indicated that maternal SeY supplementation during late gestation and lactation improved the lactation performance, redox status, and Se level transfer, and that it altered the gut microbiota in sows. In addition, maternal SeY supplementation enhanced the small intestinal barrier function and microbiota community of the weaned offspring. The results of this study will offer a reference for the function of SeY in sows’ diet and maternal–offspring integration.

As previously mentioned, when supplemented with inorganic selenite (0 to 0.3 ppm), dietary Se ranged from 0.227 to 0.651 ppm [25]. In our study, Se from basal ingredients alone provided 0.34 ppm for gestation diets and 0.37 ppm for lactation diets. These Se concentrations are greater than the NRC (2012) recommendation of 0.15 ppm, and the supplemental level frequently used in the feed industry is 0.30 ppm. In addition, many studies have indicated that maternal Se can transfer to the progeny through the placenta and milk [16,26,27]. Also, Mahan et al. [15] reported that the offspring of sows that were fed organic Se had a higher Se status both at birth and during weaning than those of sows that were fed inorganic Se. This was confirmed in the current study, where the maternal SeY intake increased the levels in the sows’ plasma and milk as well as those of the offsprings’ plasma. Consistent with a previous study, our results revealed that SeY was delivered from sow to piglet through the colostrum and milk.

Many previous studies have reported that a dietary Se source fed during late gestation and lactation had no effect on the reproductive performance of sows, including the number of pigs per litter, piglet birth weight, or litter gain from birth to weaning [15,16]. Our previous study also reported that increasing the Se supply during late gestation did not affect the reproductive performance of sows [21]. In agreement with the above, the current study indicated that a short-term increase in maternal SeY supply during late gestation did not affect the reproductive performance of sows. Nevertheless, maternal SeY supplementation during late gestation and lactation significantly increased the final average weight during the first week and the mean ADG of offspring piglets during the first week of lactation. Another study also reported that organic Se improved the ADG of suckling piglets at the first week of lactation [13]. Interestingly, we first found that the weaning-to-estrus interval was significantly shortened by SeY supplementation in sow diets. In general, 90–95% of the multiparity sows are predicted to exhibit estrus within a week after weaning. Moreover, the reduced estrus is identified as sufficient nutrient intake and body reserves [28]. Thus, SeY supplementation during late gestation and lactation might promote nutrient absorption in sows, and further nutrient digestibility experiments are needed to verify this finding. Our study indicates that short-term, it did not promote intrauterine fetal growth; however, continuous supplement during lactation increased the lactating performance of sows, including the average weight and mean ADG of piglets during the first week.

ROS overproduction during parturition and lactation may lead to oxidative stress in both the maternal pig and offspring [29]. Moreover, piglets suffer from serious oxidative stress during the newborn and weaned stages [30]. To explore the effect of maternal SeY supplementation on the antioxidant capacity transfer in parturition and lactation, we analyzed the redox status in the plasma of sows and offsprings as well as in milk. Many previous studies have jointly reported that maternal dietary organic Se supplementation during late or entire gestation could both enhance the antioxidant capacities of sows and transfer this to their offspring [13,31,32]. Similar to previous studies, our results showed that maternal SeY supplementation increased the levels of T-AOC, T-SOD, and GSH-Px, while it reduced the MDA content in the plasma and milk of sows and the plasma of their offspring. Our results verified that the maternal enhanced redox status brought about by SeY can transfer to the progeny through milk. In addition, the maternal enhanced redox status transfer was parallel with the increased Se content transfer from sows to offspring.

A previous study suggested that the immune system depends on a sufficient Se status to combat pathogens, viral invasions, and oxidative stress [33]. It has been reported that inorganic Se supplementation in a sow diet obviously increased the blood IgA, IgG, and IgM concentrations of sows and their offspring [34]. Li et al. [35] reported that organic Se addition increased the IgA and IgG in the serum of gilts. In the current study, maternal SeY supplementation during late gestation and lactation also increased the concentrations of IgA in the sow and offspring plasma and the IgG in sow milk. Similarly, other reports have found that increasing the Se intake in sows and cows during late gestation improved the immunoglobulin transfer through maternal–offspring transmission [36,37]. Our previous study also showed that increasing the organic Se supply in sows’ diet led to higher levels of plasma IgA in sows and offspring, plasma IgG in offspring, and IgA as well as IgM in milk [21]. These above results reflect that Se could enhance the passive transfer of antibodies through maternal–offspring transmission.

Furthermore, due to the antioxidant activity of selenoproteins, SeY has long been deemed to improve the intestinal barrier function through alleviating oxidative stress [38]. Proper epithelial cells are held together by the apical junctional complex, which includes transmembrane TJ proteins (claudins and occludin) and the cytosolic scaffold proteins (ZO-1) [39]. In addition, the epithelial monolayer is connected with another adheren junction, the E-cadherin-dependent barrier in the small intestine [40]. The mucus layer, an enormous polymeric network of mucins, is the first line of defense preventing the invasion of potential pathogens, and it maintains homeostasis in the small intestine [38]. Immunofluorescence analysis of MUC1 showed that maternal SeY supplementation increased the MUC1 secretion in the small intestine but did not affect the distribution in the intestinal epithelium of offspring piglets. In addition, the Western blot results showed that maternal SeY supplementation during late gestation and lactation also up-regulated the relative protein abundances of E-cadherin, ZO-1, occludin, and claudin-1 in the small intestine of weaned offspring piglets, suggesting that SeY can promote the development of intestinal barrier function in offspring piglets through mother–child interactions. Consistent with our results, Liu et al. [14] reported that adding SeY effectively improved the distribution and abundance of tight-junction protein in the jejunum. The study suggested that increased SeY addition improved intestinal barrier functions through suppressing epithelial cell apoptosis that is induced by oxidative stress [14]. Our results indicated that maternal SeY supplementation during late gestation and lactation improved the small intestinal chemical barrier and mechanical integrity of the offspring piglets.

It can be seen from previous studies that oxidative stress damages the intestinal barrier functions of the intestinal epithelial cells. Thus, whether the improved TJ protein abundance is related with the increased antioxidant capacity caused by maternal SeY supplementation was further explored. Nrf2 appears to contribute to redox homeostasis in intestinal epithelial cells [41]. Therefore, the Nrf2/Keap1 oxidative pathway was selected in order to detect the above changes in the small intestinal TJ proteins in offspring piglets. Nrf2 is a primary antioxidant transcription factor and participates in regulating the synthesis of antioxidant enzymes (MnSOD and GSH-Px) [42]. Under physiological conditions, Nrf2 is inhibited by Keap1 in the cytoplasm [43]. To combat oxidative stress, the Keap1/Nrf2 dissociation prompts the phosphorylation of Nrf2 to translocate into the nucleus and activate the transcription of an antioxidant gene [44]. Scientific evidence has proven that Nrf2-deficiency-induced oxidative stress and inflammation may indirectly impair the intestinal barrier function [45,46]. It has also been reported that SeY alleviated oxidative-stress-induced small intestinal mucosa disruption through elevating the Nrf2 expression in weaned pigs [14]. Another study found that the SeY supplementation protected chickens against the ochratoxin A-induced oxidative stress through activating in the Nrf2/Keap1 pathway [47]. The cell-cultured experiment also showed that selenomethionine alleviated oxidative stress that was induced by zearalenone via the Nrf2/Keap1 signaling pathway in IPEC-J2 cells [48]. Based on our results, maternal SeY supplementation during late gestation and lactation significantly up-regulated the phosphorylated Nrf2 and down-regulated the Keap1 protein abundances in the small intestine of weaning offspring. The activation of the Nrf2/Keap1 pathway thereby improved the redox status in offspring, which is confirmed by the results of the serum antioxidant capacity indicators.

Nutritional components are also key factors affecting the structure and abundance of the gut community structure [49]. To investigate the effects of SeY supplementation during late gestation and lactation on fecal microbiota proliferation in sows on day 1 and 21 of lactation, the microbiota compositions were analyzed using high-throughput 16S rRNA sequencing. Microbial diversity is considered to improve the stability of microbiota communities [50]. In the present study, the PCA and PLS-DA results of the beta-diversity analysis showed that the microbial community structure between the CON and SeY groups presented significant separations, suggesting that maternal SeY supplementation altered the sows’ fecal microbial community when measured on day 1 and 21 of lactation. The intestine is the harbor of numerous microbial species, belonging predominantly to the phylum Firmicutes or Bacteroidetes [51]. The present study also showed that Firmicutes and Bacteroidetes were the top two dominant phyla between the two groups. To identify the effect of dietary SeY supplement on the fecal differential bacteria in the sows on day 1 and 21 of lactation, the abundance difference and LEfSe analysis were united to analyze the differential bacterium. At the genus level, the STAMP results and LEfSe analysis together showed that dietary SeY addition increased the relative abundances of Lachnospiraceae, Prevotellaceae_UCG-001, Lachnospiraceae_NK4A136_group, and *Oscillospirales* on day 1 of lactation. *Unclassified_f_Lachnospiraceae* had been illustrated to be instrumental in producing butyrate and were associated with the inhibition of intestinal disorders [52]. In addition, to our knowledge, *Lachnospiraceae_NK4A136_group* may improve the intestinal barrier function of rats, comprises one of the main butyrate-producing bacteria, and has potentially beneficial effects on antioxidation [53,54]. *Prevotellaceae_UCG-001* belongs to the Prevotellaceae family, which has been proposed to digest amino acids and enhance calorie extraction from resistant starches, oligosaccharides, and other indigested carbohydrates [55]. In short, *unclassified_f_Lachnospiraceae*, *Prevotellaceae _UCG-001*, and *Lachnospiraceae_NK4A136_group* were all known as SCFAs producing bacteria through indigestible carbohydrates metabolization [56]. Thus, the increased SCFAs-producing bacterium might accelerate the SCFAs synthesis further to promote gut homeostasis of sows in the SeY group. Specifically, SCFAs-activated G-protein coupled receptor (GPCR) signaling has been reported to have essential roles in the colonic barrier function and anti-inflammatory and antioxidative effects in the intestine [57].

## 5. Conclusions

Taken together, these findings suggest that maternal SeY supplementation during late gestation and lactation could improve the piglets’ growth performance during the first week of lactation, Se status, antioxidant capacity, and immunoglobulin transfer, as that it could alter the fecal microbiota composition by increasing antioxidative-related and SCFAs-producing microbiota in sows. Through these mechanisms, these changes contributed to enhancing the small intestinal barrier function and activating the Nrf2/Keap1 pathway in offspring.

## Figures and Tables

**Table 1:** Effect of maternal SeY addition during late gestation and lactation on reproductive performance of sows (*n* = 35).

Item	Sow		*p*-Value
	CON	SeY	
Litters, *n*	35	35	
Total no. born per litter	11.22 ± 0.36	11.28 ± 0.29	0.906
No. of piglets born alive per litter	10.36 ± 0.33	10.53 ± 0.29	0.704
No. of stillbirths per litter	0.86 ± 0.23	0.75 ± 0.21	0.723
No. of mummies per litter	0.06 ± 0.04	0.08 ± 0.06	0.703
No. of piglets born healthy per litter	10.00 ± 0.31	10.19 ± 0.28	0.641
Litter birth weight, kg	17.51 ± 0.62	17.26 ± 0.54	0.762
Individual piglet weight, kg	1.69 ± 0.05	1.65 ± 0.03	0.473
Weight of piglets born healthy, kg	1.71 ± 0.04	1.67 ± 0.03	0.394
Stillborn weight, kg	1.25 ± 0.11	1.17 ± 0.11	0.625
Placental weight, kg	1.60 ± 0.14	1.72 ± 0.17	0.585
Placental efficiency	12.84 ± 1.18	11.71 ± 0.97	0.482
Placental weight per piglet, kg	0.11 ± 0.01	0.10 ± 0.02	0.508

CON, control group, basal diet containing 0.3 mg/kg Se as Na_2_SeO_3_; SeY, SeY group, basal diet with 0.2 mg/kg Se as Se-enriched yeast.

**Table 2:** Effect of maternal SeY addition during late gestation and lactation on growth performance of nursing piglets (*n* = 35).

Item	Sow		*p*-Value
	CON	SeY	
Litters, *n*	35	35	
Final litter size			
After day 3 of cross-fostering	9.49 ± 0.23	9.41 ± 0.27	0.835
First week	9.03 ± 0.24	8.94 ± 0.25	0.799
Second week	8.74 ± 0.25	8.74 ± 0.24	0.983
Third week	8.66 ± 0.27	8.68 ± 0.23	0.957
Preweaning survival, %	91.44 ± 2.03	92.48 ± 1.27	0.666
Final litter weight, kg			
After day 3 of cross-fostering	19.33 ± 0.67	19.38 ± 0.71	0.961
First week	28.70 ± 0.91	30.38 ± 0.98	0.213
Second week	42.49 ± 1.53	45.21 ± 1.38	0.192
Third week	58.51 ± 2.06	61.76 ± 1.78	0.283
Final average piglet weight, kg			
After day 3 of cross-fostering	2.04 ± 0.06	2.09 ± 0.08	0.622
First week	3.20 ± 0.08	3.41 ± 0.07 *	0.049
Second week	4.91 ± 0.15	5.21 ± 0.12	0.123
Third week	6.84 ± 0.21	7.18 ± 0.15	0.723
Piglet mean ADG of lactation, g/d			
First week	165.62 ± 7.22	188.31 ± 8.18 *	0.041
Second week	244.87 ± 14.08	256.88 ± 9.70	0.487
Third week	275.54 ± 12.24	280.66 ± 10.13	0.749
Mean of first to third week	228.31 ± 9.29	241.95 ± 6.64	0.239

CON, control group, basal diet containing 0.3 mg/kg Se as Na_2_SeO_3_; SeY, SeY group, basal diet with 0.2 mg/kg Se as Se-enriched yeast; * *p* < 0.05.

**Table 3:** Effects of SeY addition during late gestation and lactation on feed intake, backfat thickness, and weaning-to-estrus interval of sows (*n* = 35).

Item	Sow		*p*-Value
	CON	SeY	
ADFI of lactation, kg/d			
First week	2.98 ± 0.11	2.99 ± 0.15	0.924
Second week	4.41 ± 0.13	4.42 ± 0.16	0.967
Third week	5.13 ± 0.09	5.06 ± 0.10	0.591
Overall	4.34 ± 0.07	4.27 ± 0.09	0.572
Sow backfat thickness, mm			
Day 110 of gestation	19.54 ± 0.63	18.79 ± 0.43	0.336
Weaning	16.13 ± 0.6	15.64 ± 0.43	0.515
Loss during lactation	3.42 ± 0.28	3.15 ± 0.23	0.478
Weaning-to-estrus interval, d	5.90 ± 0.15	5.66 ± 0.11 *	0.048

CON, control group, basal diet containing 0.3 mg/kg Se as Na_2_SeO_3_; SeY, SeY group, basal diet with 0.2 mg/kg Se as Se-enriched yeast; * *p* < 0.05.

**Table 4:** Effect of SeY addition during late gestation and lactation on the colostrum and milk composition (*n* = 6).

Item	Sow		*p*-Value
	CON	SeY	
Colostrum, day 1 of lactation, %			
Fat	4.12 ± 0.31	4.20 ± 0.12	0.817
Lactose	4.92 ± 0.1	5.01 ± 0.14	0.610
Protein	7.16 ± 0.27	7.42 ± 0.54	0.682
Solids-not-fat	19.09 ± 0.73	19.71 ± 1.43	0.706
Milk, day 14 of lactation, %			
Fat	5.99 ± 0.27	6.33 ± 0.28	0.392
Lactose	6.55 ± 0.08	6.68 ± 0.23	0.615
Protein	4.73 ± 0.08	4.96 ± 0.14	0.168
Solids-not-fat	12.15 ± 0.15	12.13 ± 0.22	0.941
Milk, day 21 of lactation, %			
Fat	6.24 ± 0.09	6.91 ± 0.19 *	0.005
Lactose	6.44 ± 0.28	6.75 ± 0.21	0.392
Protein	4.63 ± 0.06	4.76 ± 0.1	0.274
Solids-not-fat	11.68 ± 0.1	11.86 ± 0.18	0.373

CON, control group, basal diet containing 0.3 mg/kg Se as Na_2_SeO_3_; SeY, SeY group, basal diet with 0.2 mg/kg Se as Se-enriched yeast; * *p* < 0.05.

**Table 5:** Effects of SeY addition during late gestation and lactation on the immune indicators of the plasma and milk of sows (*n* = 6).

Item, μg/mL	Sow		*p*-Value
	CON	SeY	
Plasma, day 1 of lactation			
IgA	658.78 ± 33.41	770.36 ± 27.08	0.022
IgG	1431.96 ± 143.16	1481.26 ± 110.87	0.789
IgM	117.79 ± 13.12	151.16 ± 23.42	0.234
Plasma, day 21 of lactation			
IgA	204.81 ± 23.96	379.31 ± 34.09 *	0.001
IgG	344.63 ± 68.34	349.58 ± 29.25	0.945
IgM	78.74 ± 10.24	79.66 ± 5.76	0.939
Colostrum, day 1 of lactation			
IgA	1002.99 ± 115.7	1246.24 ± 142.96	0.211
IgG	9793.69 ± 634.17	10,838.69 ± 576.81	0.243
IgM	973.49 ± 72.46	1098.67 ± 90.79	0.302
Milk, day 14 of lactation			
IgA	102.9 ± 6.18	121.14 ± 17.13	0.336
IgG	915.54 ± 45.43	1054.41 ± 55.30	0.076
IgM	64.53 ± 5.82	74.02 ± 7.59	0.341
Milk, day 21 of lactation			
IgA	84.89 ± 7.58	95.08 ± 7.11	0.346
IgG	801.81 ± 38.27	945.91 ± 57.60	0.059
IgM	72.42 ± 8.31	98.74 ± 12.14	0.105

CON, control group, basal diet containing 0.3 mg/kg Se as Na_2_SeO_3_; SeY, SeY group, basal diet with 0.2 mg/kg Se as Se-enriched yeast; IgA, immunoglobulin A; * *p* < 0.05.

**Table 6:** Effects of SeY supplementation during late gestation and lactation on the immune indicators in the plasma of offspring piglets (*n* = 6).

Item, μg/mL	Piglet		*p*-Value
	CON	SeY	
1 day of age			
IgA	1499.29 ± 67.03	1718.05 ± 61.05	0.036
IgG	2844.21 ± 196.39	2892.61 ± 214.82	0.871
IgM	399.49 ± 57.14	554.61 ± 50.56	0.061
21 days of age			
IgA	210.65 ± 6.90	273.06 ± 17.06	0.007
IgG	1539.68 ± 245.41	1764.78 ± 155.84	0.461
IgM	97.13 ± 10.09	111.01 ± 7.56	0.289

CON, control group, basal diet containing 0.3 mg/kg Se as Na_2_SeO_3_; SeY, SeY group, basal diet with 0.2 mg/kg Se as Se-enriched yeast; IgA, immunoglobulin A.

**Table 7:** Effects of SeY supplementation during late gestation and lactation on the antioxidative and oxidative indicators of the plasma and milk of sows (*n* = 6).

Item	Sow		*p*-Value
	CON	SeY	
Plasma, day 1 of lactation			
T-AOC, mmol/L	0.98 ± 0.03	0.97 ± 0.02	0.845
T-SOD, U/mL	59.52 ± 2.88	64.05 ± 3.64	0.287
GSH-Px, U/mL	494 ± 16.89	556.39 ± 17.75	0.229
GSH, μmol/mL	67.56 ± 1.77	69.18 ± 3.13	0.659
MDA, nmol/mL	4.00 ± 0.31	2.96 ± 0.28 *	0.028
Plasma, day 21 of lactation			
T-AOC, mmol/L	0.92 ± 0.03	1.01 ± 0.03 *	0.047
T-SOD, U/mL	47.34 ± 1.82	53.63 ± 1.29	0.104
GSH-Px, U/mL	847.88 ± 33.36	972.59 ± 22.55 *	0.011
GSH, μmol/mL	54.70 ± 2.44	58.02 ± 3.79	0.472
MDA, nmol/mL	3.17 ± 0.25	2.46 ± 0.34	0.116
Colostrum, day 1 of lactation			
T-AOC, mmol/L	1.10 ± 0.08	1.36 ± 0.07 *	0.035
T-SOD, U/mL	57.12 ± 7.76	71.96 ± 5.65	0.148
GSH-Px, U/mL	93.16 ± 10.38	124.85 ± 8.72 *	0.042
GSH, μmol/mL	11.51 ± 2.07	19.44 ± 3.11	0.055
MDA, nmol/mL	5.67 ± 0.43	3.91 ± 0.61 *	0.039
Milk, day 14 of lactation			
T-AOC, mmol/L	0.96 ± 0.06	1.09 ± 0.07	0.176
T-SOD, U/mL	52.34 ± 7.13	59.72 ± 8.78	0.526
GSH-Px, U/mL	154.9 ± 7.75	178.74 ± 7.12	0.247
GSH, μmol/mL	8.80 ± 1.63	13.1 ± 1.84	0.113
MDA, nmol/mL	4.63 ± 0.44	3.08 ± 0.31 *	0.017
Milk, day 21 of lactation			
T-AOC, mmol/L	0.89 ± 0.04	1.08 ± 0.04	0.154
T-SOD, U/mL	39.40 ± 2.97	55.48 ± 5.52 *	0.050
GSH-Px, U/mL	187.91 ± 9.31	193.41 ± 8.32	0.259
GSH, μmol/mL	8.73 ± 2.14	14.48 ± 3.55	0.190
MDA, nmol/mL	4.34 ± 0.25	3.05 ± 0.30	0.308

CON, control group, basal diet containing 0.3 mg/kg Se as Na_2_SeO_3_; SeY, SeY group, basal diet with 0.2 mg/kg Se as Se-enriched yeast; T-AOC, total antioxidant capacity; T-SOD, total superoxide dismutase; GSH-Px, glutathione peroxidase; GSH, glutathione; MDA, malondialdehyde; * *p* < 0.05.

**Table 8:** Effects of SeY supplementation during late gestation and lactation on the antioxidative and oxidative indicators of the plasma of offspring piglets (*n* = 6).

Item	Piglet		*p*-Value
	CON	SeY	
1 day of age			
T-AOC, mmol/L	0.81 ± 0.01	0.88 ± 0.08	0.375
T-SOD, U/mL	30.78 ± 1.06	34.09 ± 1.17	0.063
GSH-Px, U/mL	195.57 ± 14.29	243.75 ± 13.89 *	0.036
GSH, μmol/mL	45.98 ± 3.74	55.28 ± 4.86	0.155
MDA, nmol/mL	7.78 ± 0.46	5.70 ± 0.46 *	0.010
21 days of age			
T-AOC, mmol/L	0.79 ± 0.02	0.89 ± 0.03	0.126
T-SOD, U/mL	38.68 ± 1.85	41.89 ± 1.64	0.224
GSH-Px, U/mL	414.63 ± 20.66	460.72 ± 25.71	0.193
GSH, μmol/mL	50.9 ± 6.24	65.13 ± 1.67 *	0.048
MDA, nmol/mL	5.51 ± 0.46	4.54 ± 0.14	0.160

CON, control group, basal diet containing 0.3 mg/kg Se as Na_2_SeO_3_; SeY, SeY group, basal diet with 0.2 mg/kg Se as Se-enriched yeast; T-AOC, total antioxidant capacity; T-SOD, total superoxide dismutase; GSH-Px, glutathione peroxidase; GSH, glutathione; MDA, malondialdehyde; * *p* < 0.05.

## Data Availability

Please contact the corresponding author if further information is required.

## Supplementary Materials

The following supporting information can be downloaded at https://www.mdpi.com/article/10.3390/antiox12122064/s1: Table S1: Composition and nutrient levels of basal diets for sows (air-dry basis; %).
