# Supplementary material for: Maternal Selenium-Enriched Yeast Supplementation in Sows Enhances Offspring Growth and Antioxidant Status through the Nrf2/Keap1 Pathway
Source: Antioxidants (Basel). 2023 Dec 1;12(12):2064. doi: 10.3390/antiox12122064 (PMC10740904; doi:10.3390/antiox12122064)
Supplement: Supplementary file 1 [file antioxidants-12-02064-s001.zip › antioxidants-2733166-supplementary.pdf]

antioxidants

Article

Maternal Selenium-Enriched Yeast Supplementation in Sows Enhances Offspring Growth and Antioxidant Status through the Nrf2/Keap1 Pathway

Liang Xiong [1], Tongbin Lin [1], Xianhuai Yue [1], Shuchang Zhang [1], Xinghong Liu [1], Fang Chen [1], Shihai Zhang [1,2,3,*] and Wutai Guan [1,2,3,*]

[1] Guangdong Provincial Key Laboratory of Animal Nutrition Control, College of Animal Science, South China Agricultural University, Guangzhou 510642, China; 244628426@stu.scau.edu.cn (L.X.); 13616905519@stu.scau.edu.cn (T.L.); yxh@stu.scau.edu.cn (X.Y.); zsc0722@stu.scau.edu.cn (S.Z.); lxh@stu.scau.edu.cn (X.L.); chenfang1111@scau.edu.cn (F.C.)
[2] National Engineering Research Center for Breeding Swine Industry, South China Agricultural University, Guangzhou 510642, China
[3] Guangdong Laboratory for Lingnan Modern Agriculture, South China Agricultural University, Guangzhou 510642, China
* Correspondence: zhangshihai@scau.edu.cn (S.Z.); wtguan@scau.edu.cn (W.G.)

Table S1 Ingredient composition and nutritional levels of the basic diet (air-dried basis; %)

	Pregnant diet	Lactating diet
Ingredients		
Corn	20.50	34.10
Soybean meal	16.50	15.00
Wheat	16.00	12.00
Barley	26.90	8.00
Extruded soybean	-	20.00
Soybean sheet	15.00	3.00
Fish meal	0.80	2.50
Soybean oil	0.60	1.80
Dicalcium phosphate	1.40	1.20
Limestone	0.50	0.70
Salt	0.50	0.50
Pregnant vitamin and mineral premix[a]	1.20	
Lactating vitamin and mineral premix[b]		1.10
Mold inhibitor	0.10	0.10
Total	100.00	100.00
Nutritional levels[c]		
Digestible energy, MJ/kg	3050	3410
Metabolic energy, MJ/kg	2840	3200
Dry matter, %	87	87
Crude protein, %	13.9	17.5
Ash, %	0.76	1.1
Crude fiber, %	0.23	0.30
Crude fat, %	0.48	0.60
Calcium, %	0.53	0.72
Phosphorus, %	5.52	5.30
Available phosphorus	0.8	0.8
Digestible Lys, %c	0.6	0.6
Digestible Met + Cys	8.30	3.96
Digestible Thr, %c	2.45	5.21
Digestible Trp, %c	15	15
Se, mg/kg	0.34	0.37

[a] Pregnant vitamin and mineral premix provide per kg of complete diet: 13,000 IU vitamin A, 4000 IU vitamin D_3, 30 IU vitamin E, 4 mg vitamin K_3, 4 mg vitamin B_1, 10 mg vitamin B_2, 4.8 mg vitamin B_6, 0.034 mg vitamin B_{12}, 0.14 mg I (CaI_2O_6), 0.3 mg Se (Na_2SeO_3), 40 mg niacin, 20 mg D-pantothenate, 2.0 mg folic acid, 0.16 mg D-biotin, 80.0 mg Zn ($ZnSO_4 \cdot H_2O$), 100.0 mg Fe ($FeSO_4 \cdot H_2O$), 45.0 mg Mn ($MnSO_4 \cdot H_2O$), 15.0 mg Cu ($CuSO_4 \cdot 5H_2O$).

[b] Lactating vitamin and mineral premix provide per kg of complete diet: 13,000 IU vitamin A, 4000 IU vitamin D_3, 30 IU vitamin E, 4 mg vitamin K_3, 4 mg vitamin B_1, 10 mg vitamin B_2, 4.8 mg vitamin B_6, 0.034 mg vitamin B_{12}, 0.14 mg I (CaI_2O_6), 0.3 mg Se (Na_2SeO_3), 40 mg niacin, 20 mg D-pantothenate, 2.0 mg folic acid, 0.16 mg D-biotin, 80.0 mg Zn ($ZnSO_4 \cdot H_2O$), 100.0 mg Fe ($FeSO_4 \cdot H_2O$), 45.0 mg Mn ($MnSO_4 \cdot H_2O$), 15.0 mg Cu ($CuSO_4 \cdot 5H_2O$).

[c] Calculated values according to the tables of feed composition and nutritive values.
